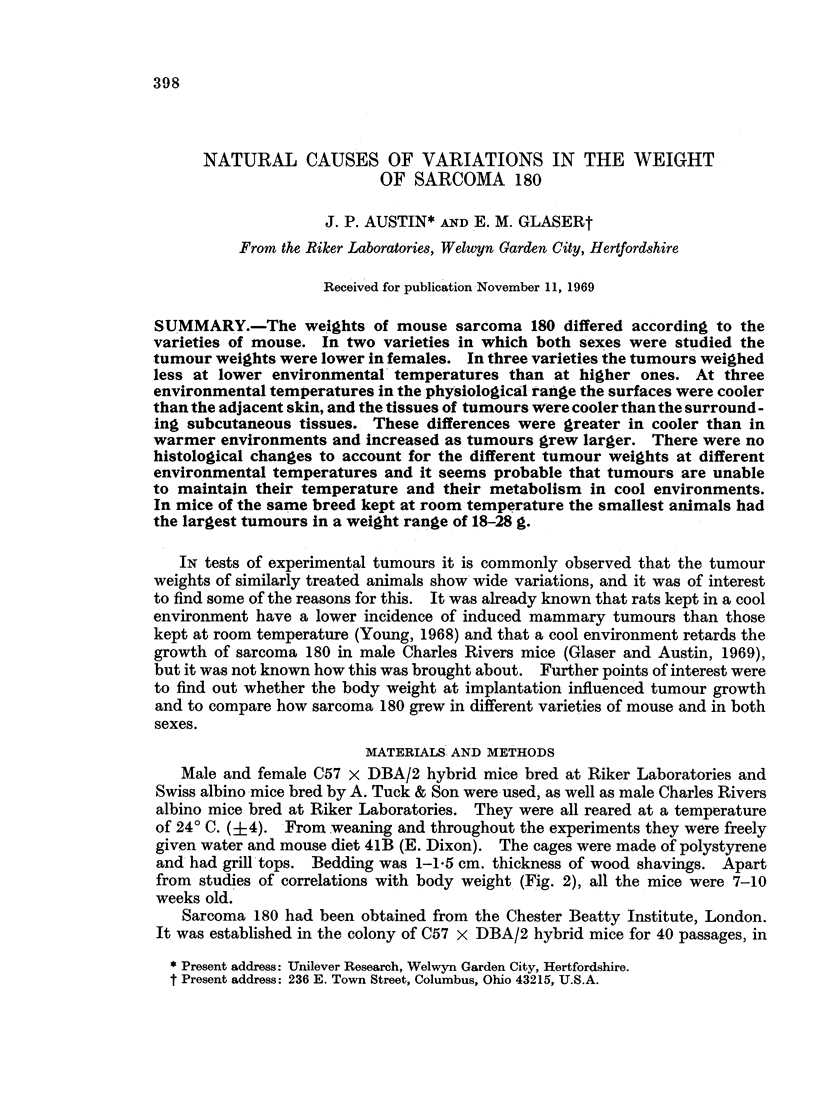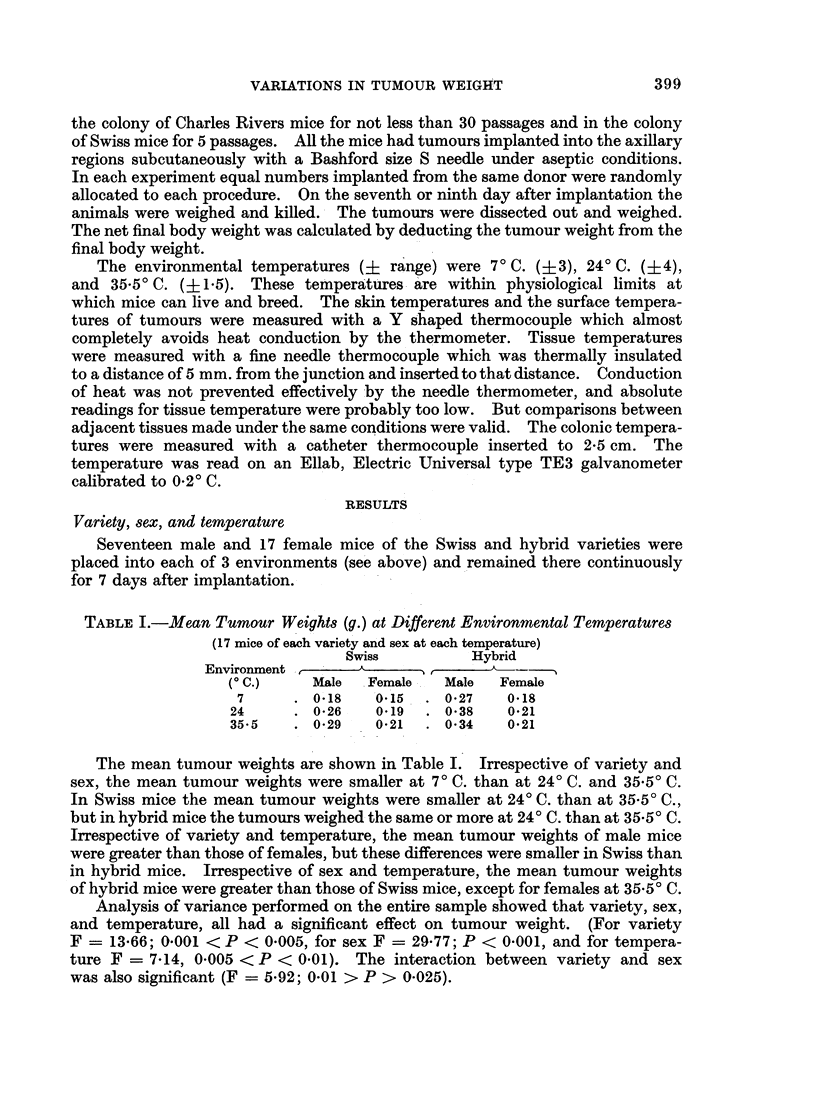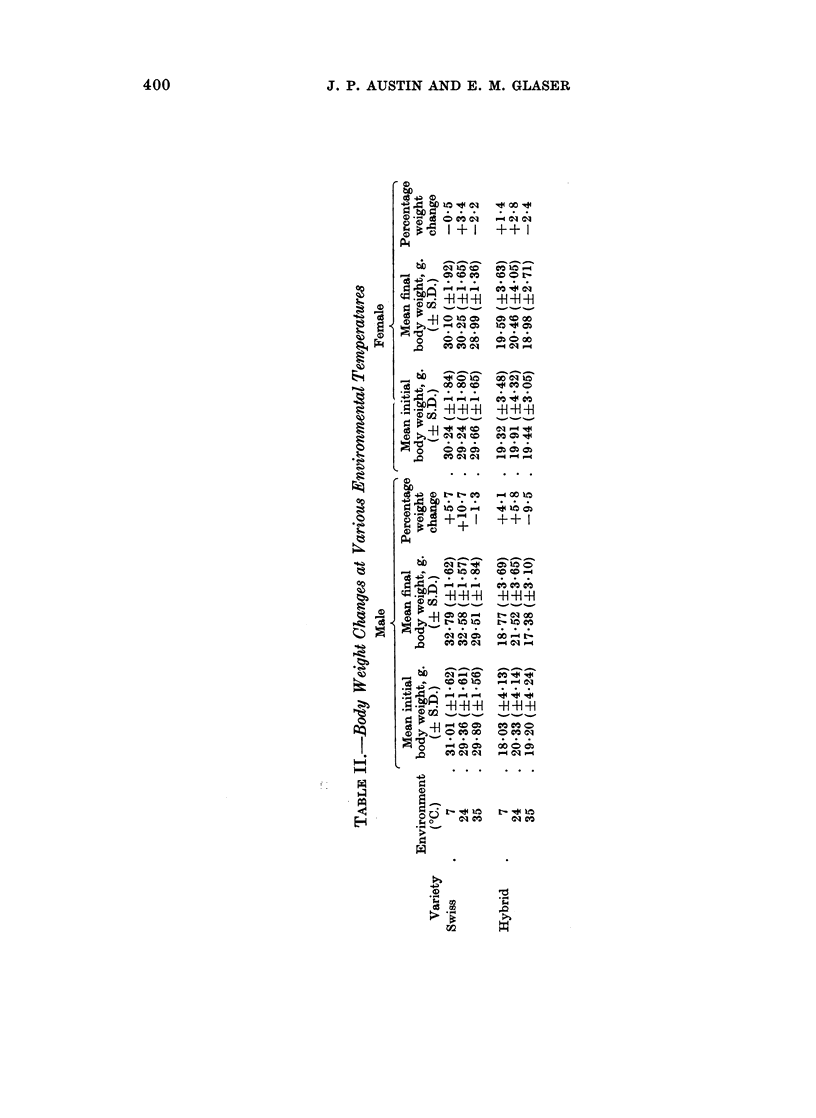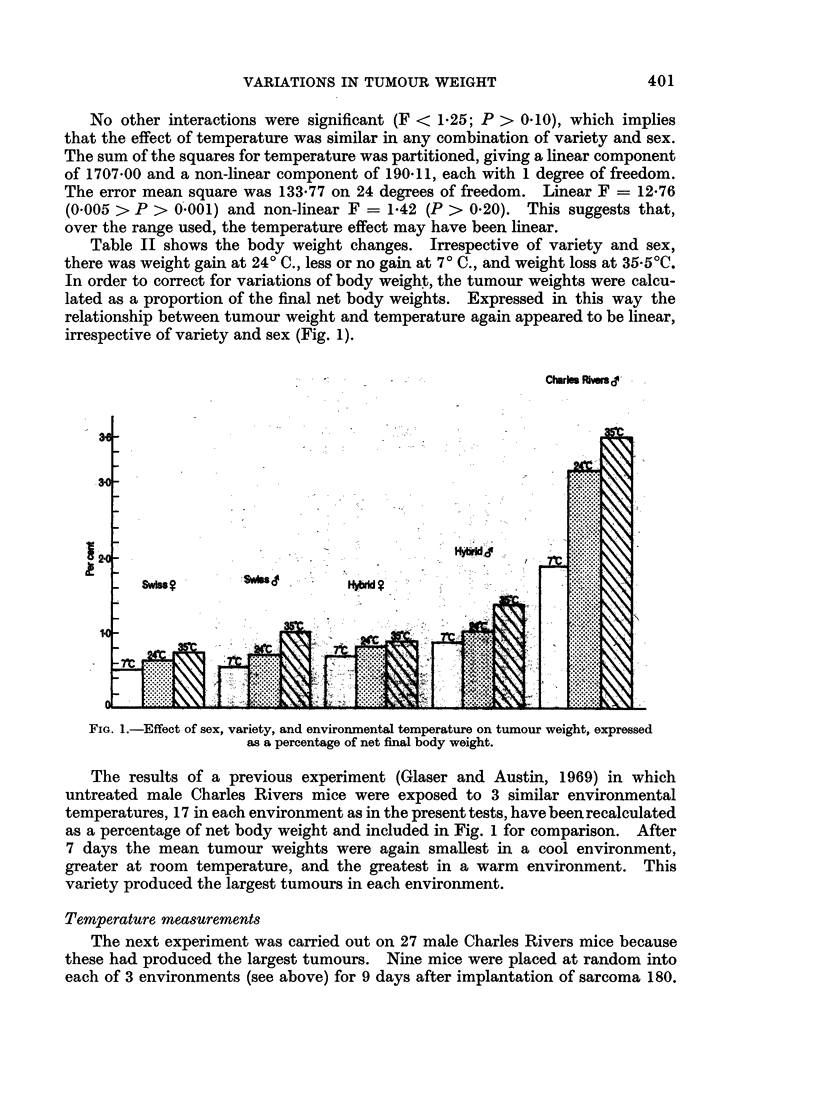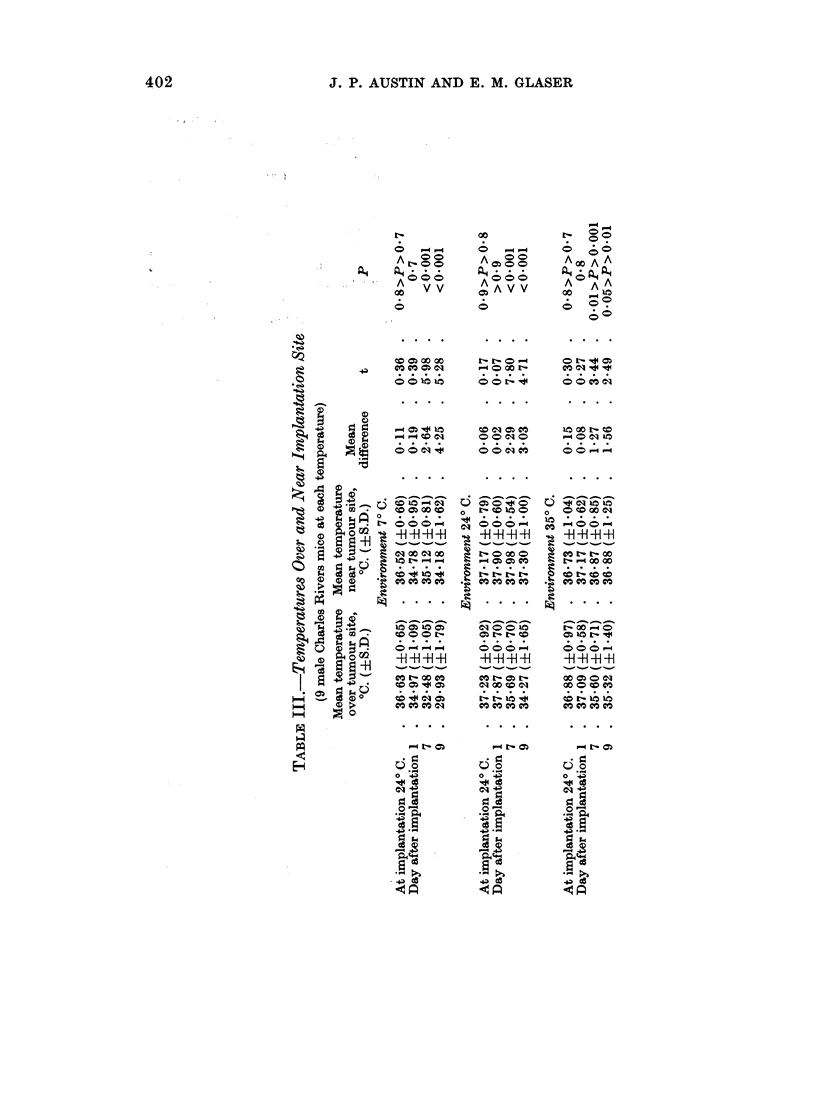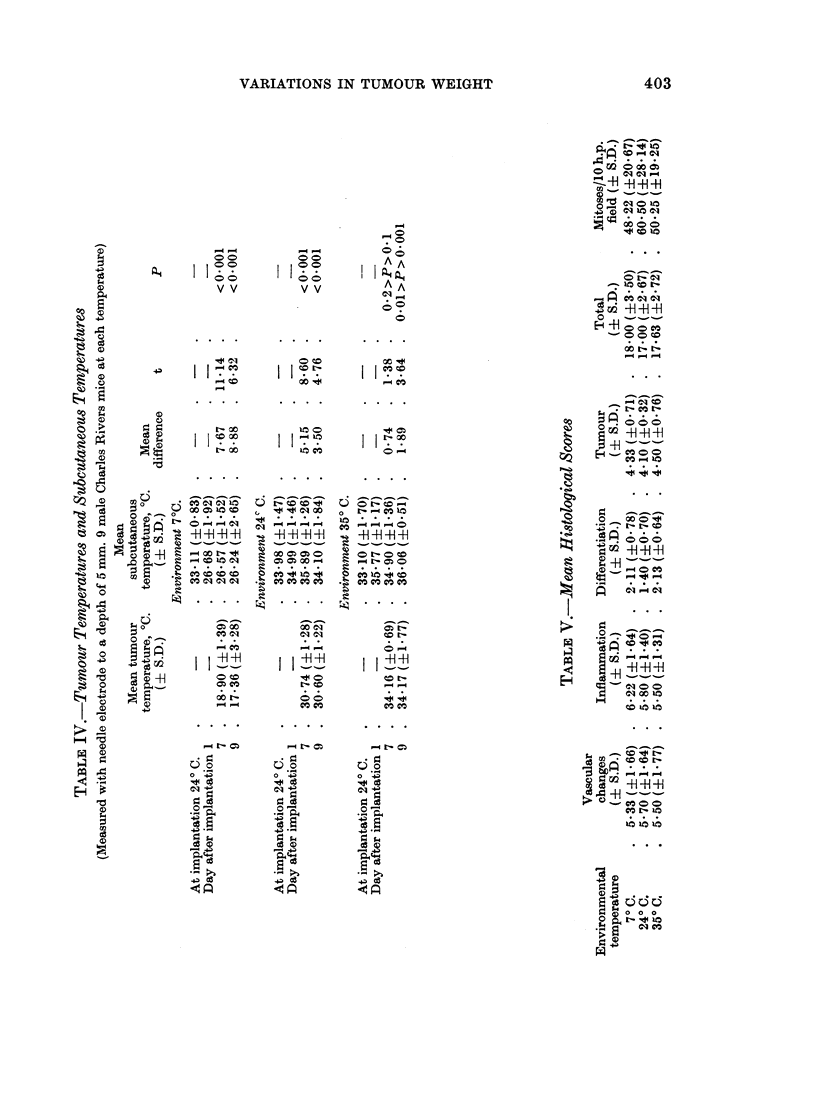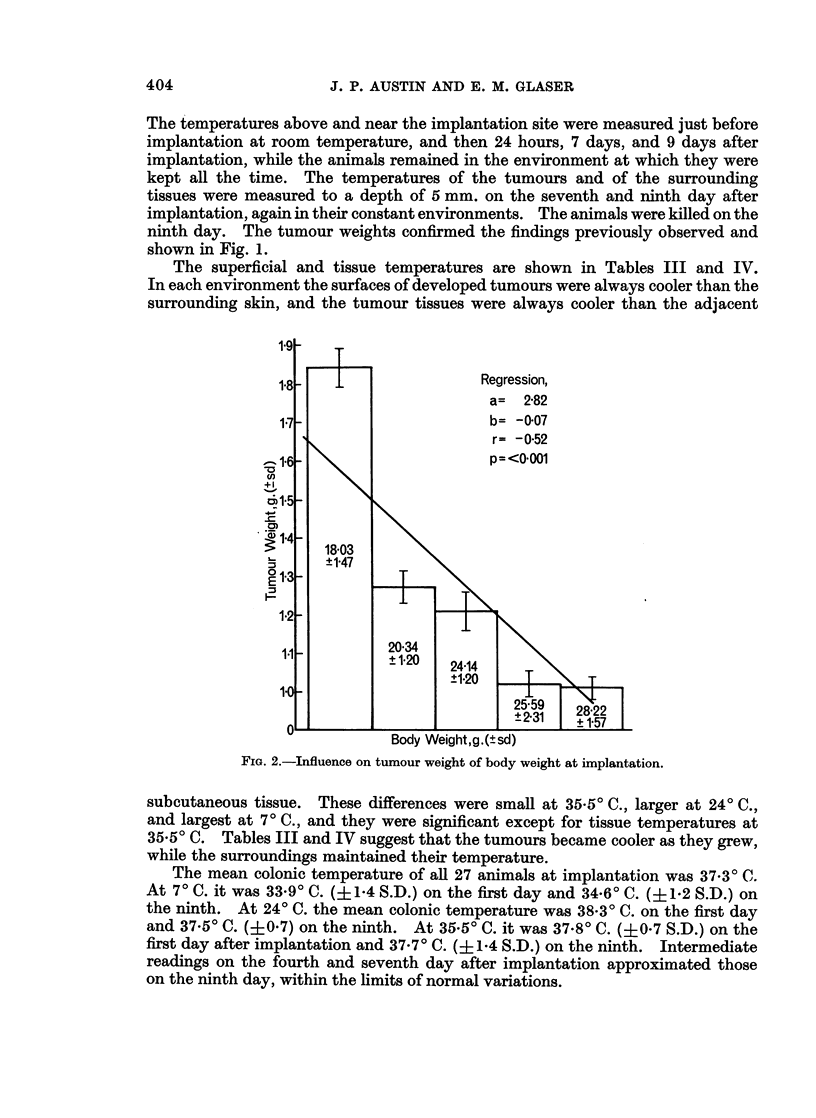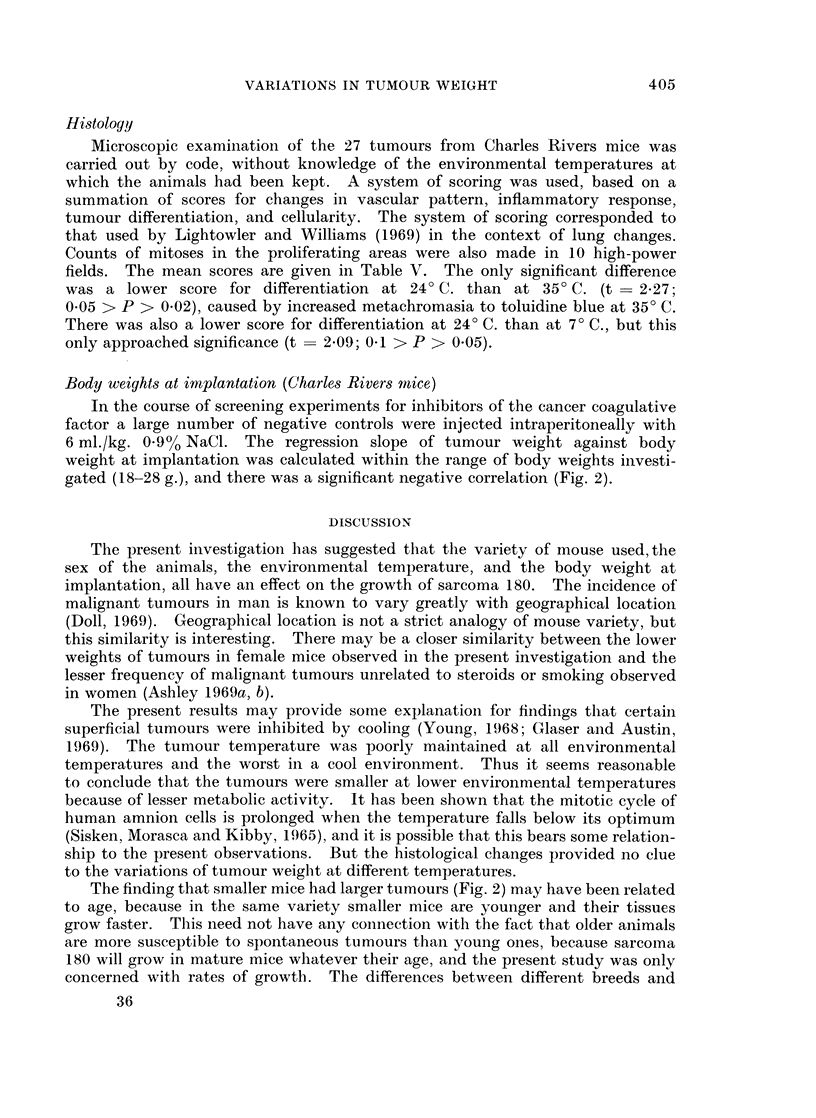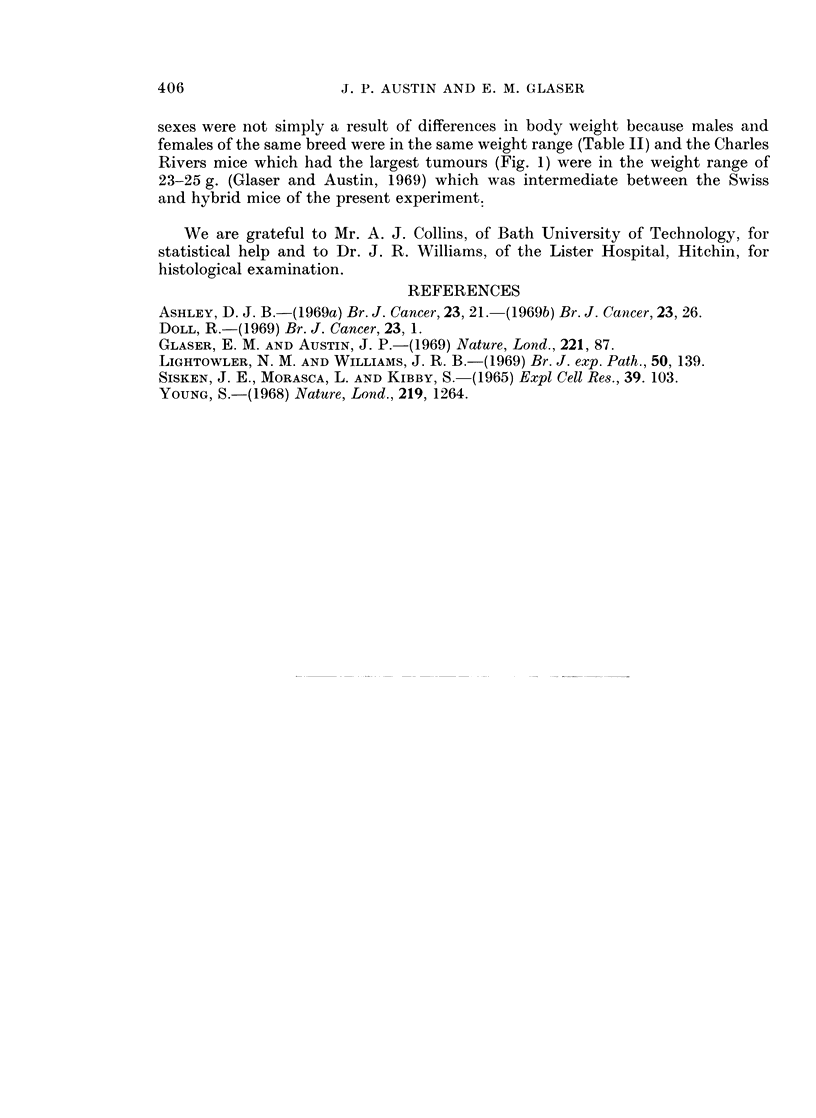# Natural Causes of Variations in the Weight of Sarcoma 180

**DOI:** 10.1038/bjc.1970.47

**Published:** 1970-06

**Authors:** J. P. Austin, E. M. Glaser

## Abstract

The weights of mouse sarcoma 180 differed according to the varieties of mouse. In two varieties in which both sexes were studied the tumour weights were lower in females. In three varieties the tumours weighed less at lower environmental temperatures than at higher ones. At three environmental temperatures in the physiological range the surfaces were cooler than the adjacent skin, and the tissues of tumours were cooler than the surrounding subcutaneous tissues. These differences were greater in cooler than in warmer environments and increased as tumours grew larger. There were no histological changes to account for the different tumour weights at different environmental temperatures and it seems probable that tumours are unable to maintain their temperature and their metabolism in cool environments. In mice of the same breed kept at room temperature the smallest animals had the largest tumours in a weight range of 18-28 g.


### NATURAL CAUSES OF VARIATIONS IN THE WEIGHT OF SARCOMA 180

J. P. AUSTIN* AND E. M. GLASER†

*From the Riker Laboratories, Welwyn Garden City, Hertfordshire*

Received for publication November 11, 1969

**SUMMARY.—The weights of mouse sarcoma 180 differed according to the varieties of mouse. In two varieties in which both sexes were studied the tumour weights were lower in females. In three varieties the tumours weighed less at lower environmental temperatures than at higher ones. At three environmental temperatures in the physiological range the surfaces were cooler than the adjacent skin, and the tissues of tumours were cooler than the surrounding subcutaneous tissues. These differences were greater in cooler than in warmer environments and increased as tumours grew larger. There were no histological changes to account for the different tumour weights at different environmental temperatures and it seems probable that tumours are unable to maintain their temperature and their metabolism in cool environments. In mice of the same breed kept at room temperature the smallest animals had the largest tumours in a weight range of 18–28 g.**

IN tests of experimental tumours it is commonly observed that the tumour weights of similarly treated animals show wide variations, and it was of interest to find some of the reasons for this. It was already known that rats kept in a cool environment have a lower incidence of induced mammary tumours than those kept at room temperature (Young, 1968) and that a cool environment retards the growth of sarcoma 180 in male Charles Rivers mice (Glaser and Austin, 1969), but it was not known how this was brought about. Further points of interest were to find out whether the body weight at implantation influenced tumour growth and to compare how sarcoma 180 grew in different varieties of mouse and in both sexes.

### MATERIALS AND METHODS

Male and female C57 × DBA/2 hybrid mice bred at Riker Laboratories and Swiss albino mice bred by A. Tuck & Son were used, as well as male Charles Rivers albino mice bred at Riker Laboratories. They were all reared at a temperature of 24° C. (±4). From weaning and throughout the experiments they were freely given water and mouse diet 41B (E. Dixon). The cages were made of polystyrene and had grill tops. Bedding was 1–1·5 cm. thickness of wood shavings. Apart from studies of correlations with body weight (Fig. 2), all the mice were 7–10 weeks old.

Sarcoma 180 had been obtained from the Chester Beatty Institute, London. It was established in the colony of C57 × DBA/2 hybrid mice for 40 passages, in

* Present address: Unilever Research, Welwyn Garden City, Hertfordshire.
† Present address: 236 E. Town Street, Columbus, Ohio 43215, U.S.A.

the colony of Charles Rivers mice for not less than 30 passages and in the colony of Swiss mice for 5 passages. All the mice had tumours implanted into the axillary regions subcutaneously with a Bashford size S needle under aseptic conditions. In each experiment equal numbers implanted from the same donor were randomly allocated to each procedure. On the seventh or ninth day after implantation the animals were weighed and killed. The tumours were dissected out and weighed. The net final body weight was calculated by deducting the tumour weight from the final body weight.

The environmental temperatures (± range) were 7° C. (±3), 24° C. (±4), and 35·5° C. (±1·5). These temperatures are within physiological limits at which mice can live and breed. The skin temperatures and the surface temperatures of tumours were measured with a Y shaped thermocouple which almost completely avoids heat conduction by the thermometer. Tissue temperatures were measured with a fine needle thermocouple which was thermally insulated to a distance of 5 mm. from the junction and inserted to that distance. Conduction of heat was not prevented effectively by the needle thermometer, and absolute readings for tissue temperature were probably too low. But comparisons between adjacent tissues made under the same conditions were valid. The colonic temperatures were measured with a catheter thermocouple inserted to 2·5 cm. The temperature was read on an Ellab, Electric Universal type TE3 galvanometer calibrated to 0·2° C.

### RESULTS

### *Variety, sex, and temperature*

Seventeen male and 17 female mice of the Swiss and hybrid varieties were placed into each of 3 environments (see above) and remained there continuously for 7 days after implantation.

TABLE I.—*Mean Tumour Weights (g.) at Different Environmental Temperatures*

(17 mice of each variety and sex at each temperature)

| Environment (° C.) | Swiss Male | Swiss Female | Hybrid Male | Hybrid Female |
|---|---|---|---|---|
| 7 | 0·18 | 0·15 | 0·27 | 0·18 |
| 24 | 0·26 | 0·19 | 0·38 | 0·21 |
| 35·5 | 0·29 | 0·21 | 0·34 | 0·21 |

The mean tumour weights are shown in Table I. Irrespective of variety and sex, the mean tumour weights were smaller at 7° C. than at 24° C. and 35·5° C. In Swiss mice the mean tumour weights were smaller at 24° C. than at 35·5° C., but in hybrid mice the tumours weighed the same or more at 24° C. than at 35·5° C. Irrespective of variety and temperature, the mean tumour weights of male mice were greater than those of females, but these differences were smaller in Swiss than in hybrid mice. Irrespective of sex and temperature, the mean tumour weights of hybrid mice were greater than those of Swiss mice, except for females at 35·5° C.

Analysis of variance performed on the entire sample showed that variety, sex, and temperature, all had a significant effect on tumour weight. (For variety $F = 13{\cdot}66$; $0{\cdot}001 < P < 0{\cdot}005$, for sex $F = 29{\cdot}77$; $P < 0{\cdot}001$, and for temperature $F = 7{\cdot}14$, $0{\cdot}005 < P < 0{\cdot}01$). The interaction between variety and sex was also significant ($F = 5{\cdot}92$; $0{\cdot}01 > P > 0{\cdot}025$).

TABLE II.—*Body Weight Changes at Various Environmental Temperatures*

| Variety | Environment (°C.) | Male: Mean initial body weight, g. (± S.D.) | Male: Mean final body weight, g. (± S.D.) | Male: Percentage weight change | Female: Mean initial body weight, g. (± S.D.) | Female: Mean final body weight, g. (± S.D.) | Female: Percentage weight change |
|---|---|---|---|---|---|---|---|
| Swiss | 7 | 31·01 (±1·62) | 32·79 (±1·62) | +5·7 | 30·24 (±1·84) | 30·10 (±1·92) | −0·5 |
| | 24 | 29·36 (±1·61) | 32·58 (±1·57) | +10·7 | 29·24 (±1·80) | 30·25 (±1·65) | +3·4 |
| | 35 | 29·89 (±1·56) | 29·51 (±1·84) | −1·3 | 29·66 (±1·65) | 28·99 (±1·36) | −2·2 |
| Hybrid | 7 | 18·03 (±4·13) | 18·77 (±3·69) | +4·1 | 19·32 (±3·48) | 19·59 (±3·63) | +1·4 |
| | 24 | 20·33 (±4·14) | 21·52 (±3·65) | +5·8 | 19·91 (±4·32) | 20·46 (±4·05) | +2·8 |
| | 35 | 19·20 (±4·24) | 17·38 (±3·10) | −9·5 | 19·44 (±3·05) | 18·98 (±2·71) | −2·4 |

No other interactions were significant ($F < 1{\cdot}25$; $P > 0{\cdot}10$), which implies that the effect of temperature was similar in any combination of variety and sex. The sum of the squares for temperature was partitioned, giving a linear component of 1707·00 and a non-linear component of 190·11, each with 1 degree of freedom. The error mean square was 133·77 on 24 degrees of freedom. Linear $F = 12{\cdot}76$ ($0{\cdot}005 > P > 0{\cdot}001$) and non-linear $F = 1{\cdot}42$ ($P > 0{\cdot}20$). This suggests that, over the range used, the temperature effect may have been linear.

Table II shows the body weight changes. Irrespective of variety and sex, there was weight gain at 24° C., less or no gain at 7° C., and weight loss at 35·5°C. In order to correct for variations of body weight, the tumour weights were calculated as a proportion of the final net body weights. Expressed in this way the relationship between tumour weight and temperature again appeared to be linear, irrespective of variety and sex (Fig. 1).

FIG. 1.—Effect of sex, variety, and environmental temperature on tumour weight, expressed as a percentage of net final body weight.

The results of a previous experiment (Glaser and Austin, 1969) in which untreated male Charles Rivers mice were exposed to 3 similar environmental temperatures, 17 in each environment as in the present tests, have been recalculated as a percentage of net body weight and included in Fig. 1 for comparison. After 7 days the mean tumour weights were again smallest in a cool environment, greater at room temperature, and the greatest in a warm environment. This variety produced the largest tumours in each environment.

*Temperature measurements*

The next experiment was carried out on 27 male Charles Rivers mice because these had produced the largest tumours. Nine mice were placed at random into each of 3 environments (see above) for 9 days after implantation of sarcoma 180.

TABLE III.—*Temperatures Over and Near Implantation Site*

(9 male Charles Rivers mice at each temperature)

| | Mean temperature over tumour site, °C. (±S.D.) | Mean temperature near tumour site, °C. (±S.D.) | Mean difference | t | P |
|---|---|---|---|---|---|
| | | *Environment* 7° C. | | | |
| At implantation 24° C. | 36·63 (±0·65) | 36·52 (±0·66) | 0·11 | 0·36 | 0·8>$P$>0·7 |
| Day after implantation 1 | 34·97 (±1·09) | 34·78 (±0·95) | 0·19 | 0·39 | 0·7 |
| 7 | 32·48 (±1·05) | 35·12 (±0·81) | 2·64 | 5·98 | <0·001 |
| 9 | 29·93 (±1·79) | 34·18 (±1·62) | 4·25 | 5·28 | <0·001 |
| | | *Environment* 24° C. | | | |
| At implantation 24° C. | 37·23 (±0·92) | 37·17 (±0·79) | 0·06 | 0·17 | 0·9>$P$>0·8 |
| Day after implantation 1 | 37·87 (±0·70) | 37·90 (±0·60) | 0·02 | 0·07 | >0·9 |
| 7 | 35·69 (±0·70) | 37·98 (±0·54) | 2·29 | 7·80 | <0·001 |
| 9 | 34·27 (±1·65) | 37·30 (±1·00) | 3·03 | 4·71 | <0·001 |
| | | *Environment* 35° C. | | | |
| At implantation 24° C. | 36·88 (±0·97) | 36·73 (±1·04) | 0·15 | 0·30 | 0·8>$P$>0·7 |
| Day after implantation 1 | 37·09 (±0·58) | 37·17 (±0·62) | 0·08 | 0·27 | 0·8 |
| 7 | 35·60 (±0·71) | 36·87 (±0·85) | 1·27 | 3·44 | 0·01>$P$>0·001 |
| 9 | 35·32 (±1·40) | 36·88 (±1·25) | 1·56 | 2·49 | 0·05>$P$>0·01 |

TABLE IV.—*Tumour Temperatures and Subcutaneous Temperatures*

(Measured with needle electrode to a depth of 5 mm. 9 male Charles Rivers mice at each temperature)

| | Mean tumour temperature, °C. (± S.D.) | Mean subcutaneous temperature, °C. (± S.D.) | Mean difference | t | P |
|---|---|---|---|---|---|
| | | *Environment* 7 °C. | | | |
| At implantation 24° C. | — | 33·11 (±0·83) | — | — | — |
| Day after implantation 1 | — | 26·68 (±1·92) | — | — | — |
| 7 | 18·90 (±1·39) | 26·57 (±1·52) | 7·67 | 11·14 | $<0·001$ |
| 9 | 17·36 (±3·28) | 26·24 (±2·65) | 8·88 | 6·32 | $<0·001$ |
| | | *Environment* 24° C. | | | |
| At implantation 24° C. | — | 33·98 (±1·47) | — | — | — |
| Day after implantation 1 | — | 34·99 (±1·46) | — | — | — |
| 7 | 30·74 (±1·28) | 35·89 (±1·26) | 5·15 | 8·60 | $<0·001$ |
| 9 | 30·60 (±1·22) | 34·10 (±1·84) | 3·50 | 4·76 | $<0·001$ |
| | | *Environment* 35° C. | | | |
| At implantation 24° C. | — | 33·10 (±1·70) | — | — | — |
| Day after implantation 1 | — | 35·77 (±1·17) | — | — | — |
| 7 | 34·16 (±0·69) | 34·90 (±1·36) | 0·74 | 1·38 | $0·2>P>0·1$ |
| 9 | 34·17 (±1·77) | 36·06 (±0·51) | 1·89 | 3·64 | $0·01>P>0·001$ |

TABLE V.—*Mean Histological Scores*

| Environmental temperature | Vascular changes (± S.D.) | Inflammation (± S.D.) | Differentiation (± S.D.) | Tumour (± S.D.) | Total (± S.D.) | Mitoses/10 h.p. field (± S.D.) |
|---|---|---|---|---|---|---|
| 7° C. | 5·33 (±1·66) | 6·22 (±1·64) | 2·11 (±0·78) | 4·33 (±0·71) | 18·00 (±3·50) | 48·22 (±20·67) |
| 24° C. | 5·70 (±1·64) | 5·80 (±1·40) | 1·40 (±0·70) | 4·10 (±0·32) | 17·00 (±2·67) | 60·50 (±28·14) |
| 35° C. | 5·50 (±1·77) | 5·50 (±1·31) | 2·13 (±0·64) | 4·50 (±0·76) | 17·63 (±2·72) | 50·25 (±19·25) |

The temperatures above and near the implantation site were measured just before implantation at room temperature, and then 24 hours, 7 days, and 9 days after implantation, while the animals remained in the environment at which they were kept all the time. The temperatures of the tumours and of the surrounding tissues were measured to a depth of 5 mm. on the seventh and ninth day after implantation, again in their constant environments. The animals were killed on the ninth day. The tumour weights confirmed the findings previously observed and shown in Fig. 1.

The superficial and tissue temperatures are shown in Tables III and IV. In each environment the surfaces of developed tumours were always cooler than the surrounding skin, and the tumour tissues were always cooler than the adjacent

Fig. 2.—Influence on tumour weight of body weight at implantation.

subcutaneous tissue. These differences were small at 35·5° C., larger at 24° C., and largest at 7° C., and they were significant except for tissue temperatures at 35·5° C. Tables III and IV suggest that the tumours became cooler as they grew, while the surroundings maintained their temperature.

The mean colonic temperature of all 27 animals at implantation was 37·3° C. At 7° C. it was 33·9° C. (±1·4 S.D.) on the first day and 34·6° C. (±1·2 S.D.) on the ninth. At 24° C. the mean colonic temperature was 38·3° C. on the first day and 37·5° C. (±0·7) on the ninth. At 35·5° C. it was 37·8° C. (±0·7 S.D.) on the first day after implantation and 37·7° C. (±1·4 S.D.) on the ninth. Intermediate readings on the fourth and seventh day after implantation approximated those on the ninth day, within the limits of normal variations.

*Histology*

Microscopic examination of the 27 tumours from Charles Rivers mice was carried out by code, without knowledge of the environmental temperatures at which the animals had been kept. A system of scoring was used, based on a summation of scores for changes in vascular pattern, inflammatory response, tumour differentiation, and cellularity. The system of scoring corresponded to that used by Lightowler and Williams (1969) in the context of lung changes. Counts of mitoses in the proliferating areas were also made in 10 high-power fields. The mean scores are given in Table V. The only significant difference was a lower score for differentiation at 24° C. than at 35° C. ($t = 2{\cdot}27$; $0{\cdot}05 > P > 0{\cdot}02$), caused by increased metachromasia to toluidine blue at 35° C. There was also a lower score for differentiation at 24° C. than at 7° C., but this only approached significance ($t = 2{\cdot}09$; $0{\cdot}1 > P > 0{\cdot}05$).

*Body weights at implantation (Charles Rivers mice)*

In the course of screening experiments for inhibitors of the cancer coagulative factor a large number of negative controls were injected intraperitoneally with 6 ml./kg. 0·9% NaCl. The regression slope of tumour weight against body weight at implantation was calculated within the range of body weights investigated (18–28 g.), and there was a significant negative correlation (Fig. 2).

### DISCUSSION

The present investigation has suggested that the variety of mouse used, the sex of the animals, the environmental temperature, and the body weight at implantation, all have an effect on the growth of sarcoma 180. The incidence of malignant tumours in man is known to vary greatly with geographical location (Doll, 1969). Geographical location is not a strict analogy of mouse variety, but this similarity is interesting. There may be a closer similarity between the lower weights of tumours in female mice observed in the present investigation and the lesser frequency of malignant tumours unrelated to steroids or smoking observed in women (Ashley 1969*a*, *b*).

The present results may provide some explanation for findings that certain superficial tumours were inhibited by cooling (Young, 1968; Glaser and Austin, 1969). The tumour temperature was poorly maintained at all environmental temperatures and the worst in a cool environment. Thus it seems reasonable to conclude that the tumours were smaller at lower environmental temperatures because of lesser metabolic activity. It has been shown that the mitotic cycle of human amnion cells is prolonged when the temperature falls below its optimum (Sisken, Morasca and Kibby, 1965), and it is possible that this bears some relationship to the present observations. But the histological changes provided no clue to the variations of tumour weight at different temperatures.

The finding that smaller mice had larger tumours (Fig. 2) may have been related to age, because in the same variety smaller mice are younger and their tissues grow faster. This need not have any connection with the fact that older animals are more susceptible to spontaneous tumours than young ones, because sarcoma 180 will grow in mature mice whatever their age, and the present study was only concerned with rates of growth. The differences between different breeds and sexes were not simply a result of differences in body weight because males and females of the same breed were in the same weight range (Table II) and the Charles Rivers mice which had the largest tumours (Fig. 1) were in the weight range of 23–25 g. (Glaser and Austin, 1969) which was intermediate between the Swiss and hybrid mice of the present experiment.

We are grateful to Mr. A. J. Collins, of Bath University of Technology, for statistical help and to Dr. J. R. Williams, of the Lister Hospital, Hitchin, for histological examination.

### REFERENCES

Ashley, D. J. B.—(1969*a*) *Br. J. Cancer*, **23**, 21.—(1969*b*) *Br. J. Cancer*, **23**, 26.
Doll, R.—(1969) *Br. J. Cancer*, **23**, 1.
Glaser, E. M. and Austin, J. P.—(1969) *Nature, Lond.*, **221**, 87.
Lightowler, N. M. and Williams, J. R. B.—(1969) *Br. J. exp. Path.*, **50**, 139.
Sisken, J. E., Morasca, L. and Kibby, S.—(1965) *Expl Cell Res.*, **39**. 103.
Young, S.—(1968) *Nature, Lond.*, **219**, 1264.